# Immunosignatures associated with *TP53* status and co-mutations classify prognostically head and neck cancer patients

**DOI:** 10.1186/s12943-023-01905-9

**Published:** 2023-11-30

**Authors:** Andrea Sacconi, Paola Muti, Claudio Pulito, Giulia Urbani, Matteo Allegretti, Raul Pellini, Nikolay Mehterov, Uri Ben-David, Sabrina Strano, Paolo Bossi, Giovanni Blandino

**Affiliations:** 1 Clinical Trial Center, Biostatistics and Bioinformatics, IRCCS Regina Elena National Cancer Institute, Rome, 00144 Italy; 2 Department of Biomedical, Surgical Science and Oral Health, Milan University, Milan, Italy; 3 Fondazione IRCCS Ca’ Granda Ospedale Maggiore Policlinico, Maxillo-Facial Surgery and Dental Unit, Milan, Italy; 4 Translational Oncology Research Unit, IRCCS Regina Elena National Cancer Institute, Rome, 00144 Italy; 5 Otolaryngology Head and Neck Surgery Unit, IRCCS Regina Elena National Cancer Institute, Rome, Italy; 6 Department of Medical Biology, Medical University-Plovdiv, Plodvid, Bulgaria; 7 Research Institute, Medical University-Plovdiv, Plovdiv, Bulgaria; 8 Department of Human Molecular Genetics and Biochemistry, Faculty of Medicine, Tel Aviv University, Tel Aviv, Israel; 9 SAFU Unit, IRCCS Regina Elena National Cancer Institute, 00144 Rome, Italy; 10 Department of Biomedical Sciences, Humanitas University, Via Rita Levi Montalcini 4, 20072 Pieve Emanuele, Milan, Italy; 11 IRCCS Humanitas Research Hospital, Via Manzoni 56, 20089 Rozzano, Milan, Italy

**Keywords:** HNSCC, Immunotherapy, Immune checkpoint inhibitor, p53, PDL1, c-MYC, PI3K

## Abstract

**Background:**

Immune checkpoint inhibitors (ICIs) are a therapeutic strategy for various cancers although only a subset of patients respond to the therapy. Identifying patients more prone to respond to ICIs may increase the therapeutic benefit and allow studying new approaches for resistant patients.

**Methods:**

We analyzed the TCGA cohort of HNSCC patients in relation to their activation of 26 immune gene expression signatures, as well as their cell type composition, in order to define signaling pathways associated with resistance to ICIs. Results were validated on two cohorts of 102 HNSCC patients and 139 HNSCC patients under treatment with PD-L1 inhibitors, respectively, and a cohort of 108 HNSCC HPV negative patients and by in vitro experiments in HNSCC cell lines.

**Results:**

We observed a significant association between the gene set and *TP53* gene status and OS and PFS of HNSCC patients. Surprisingly, the presence of a *TP53* mutation together with another co-driver mutation was associated with significantly higher levels of the immune gene expression, in comparison to tumors in which the *TP53* gene was mutated alone. In addition, the higher level of *TP53* mutated-dependent MYC signature was associated with lower levels of the immune gene expression signature. In vitro and three different patient cohorts validation analyses corroborated these findings.

**Conclusions:**

Immune gene signature sets associated with TP53 status and co-mutations classify with more accuracy HNSCC patients. These biomarkers may be easily implemented in clinical setting.

**Supplementary Information:**

The online version contains supplementary material available at 10.1186/s12943-023-01905-9.

## Background

Every year, almost one million people are affected by Head and Neck Squamous Cell Carcinoma (HNSCC) in the world [1]. HNSCC is a biologically diverse and genomically heterogeneous disease that emerges from the squamous mucosal lining of the upper aerodigestive tract, including the lip and oral cavity, nasal cavity, paranasal sinuses, nasopharynx, oropharynx, larynx and hypopharynx [2]. Most patients present with a locally advanced disease with a high risk of recurrence, and approximately 10% of them presents with a metastatic disease [3]. The 5-year survival for HNSCC patients across all stages is ~ 40%–50% [4], while the median overall survival for recurrent/metastatic (R-M) patients is 10–14 months only [5]. Immune checkpoint inhibitors (ICIs) are an active category of immunotherapies that block inhibitory immune checkpoint pathways in order to reactivate immune responses against cancer. In 2016, the US Food and Drug Administration (FDA) approved two ICIs, the anti-programmed cell death protein (PD-1) monoclonal antibodies, nivolumab (Opdivo, Bristol-Myers Squibb) and pembrolizumab (Keytruda, Merck), for the treatment of R-M HNSCC patients refractory to platinum-based therapy. The same agency then approved pembrolizumab for the first-line treatment of patients with unresectable R-M HNSCC. Understanding what determines the response of HNSCC to ICIs is therefore of utmost clinical importance.

The use of immune checkpoint inhibitors (ICIs) is increasing in several cancer settings, both as monotherapy and as combinations with another ICI, chemotherapy, or targeted agents. The benefit of this new class of drugs seems to be large but limited to a subgroup of patients, thus an efficient patient characterization is needed to guide improvements in treatment. Predictors of response to ICIs are critical to ensure optimal selection of patients to be offered these drugs, thus achieving higher response, preserving patients from unnecessary toxicities, and saving economic resources. Also, this could allow an early activation of other therapeutic strategies, including clinical trials, for patients whose probability of response to ICIs is predicted to be very low. However, there are currently very few markers available, the most important of which are PDL1, microsatellite instability and tumour mutational burden (TMB) assessment; of these, only the assessment of PDL1 as a combined positive score (CPS) is utilized as a predictive marker in first-line R-M HNSCC. Several clinical, molecular, and microbiological factors are assumed to have a role as influencing response to ICIs. Recently, the ASCO and the College of American Pathologists discussed the challenges and opportunities of using biomarkers to predict the benefit from ICI, underlying the need for a more comprehensive evaluation, including testing of group of biomarkers, standardizing assays and generating more data to address the open questions [6].

The aim of the present report is to assess the role of *TP53* gene status together with co-driver mutations as prognostic predictors for classifying HNSCC patients, adjusting for potential confounders. We applied a bioinformatic approach and validated the results in experimental studies with human cancer cell lines and in analyses of three independent cohorts of HNSCC patients.

## Results

The study was conducted to investigate the association between 26 immune gene sets (listed in Table S1) [7] and immune checkpoint proteins expressed in HNSCC and their effect on patient survival. In the current study, we assessed these signatures in HNSCC, as 20 of the 26 gene sets were also found to be modulated in HNSCC [8]. In particular, in the present analysis we assessed the effect of the 26 immunosignature on both the programmed cell death protein ligand-1 (PD-L1) axis and the T-lymphocyte associated protein 4 (CTLA-4) axis, controlling for potential confounders and effect modifiers [9]. In the 520 participants of the TCGA the variables relating to the characteristics of the patients showed how adulthood, between 53 and 69 years, was the one with the highest prevalence with 48% of cases represented in that range as well as the male versus female gender with 74% of the cases considered. The distribution by tumor volume showed similar frequency between T2, T3 and T4 (29%, 26% and 35%, respectively), as well as the nodal status with 47% for N0 50% for N + . When we looked at the disease stage, stage IV represented half of the observations, while stage II and III were equally distributed around 20% each and stage I, instead, showed the smallest frequency with 4% of the observations. As expected, HPV negative cases were the vast majority of cases compared to positive cases (81% versus 19%) as well as alcohol consumers (67%) versus no-alcohol consumers. Active smokers showed a slightly higher frequency than non-smokers (34% versus 22%) and ex-smokers who quitted more recently (less than 15 years) showed a higher frequency of cancers than those who stopped smoking for a longer period of time (greater than 15 years). As regards the mutational characteristics, lesions with TP53 mutations were more frequent than those without mutation (70%) while the mutated forms of CDKN2A, FAT1 and PIK3CA showed a lower frequency of mutated forms than those non-mutated with 78% of non-mutated cases for CDKN2A, FAT1 and 82% for PIK3CA (Table S2). The 26 immune gene expression signatures, the related analysis of the genes that comprise these signatures, and the analysis of the 15 immune cell types, are described in Table S1.

**Fig. 1:**
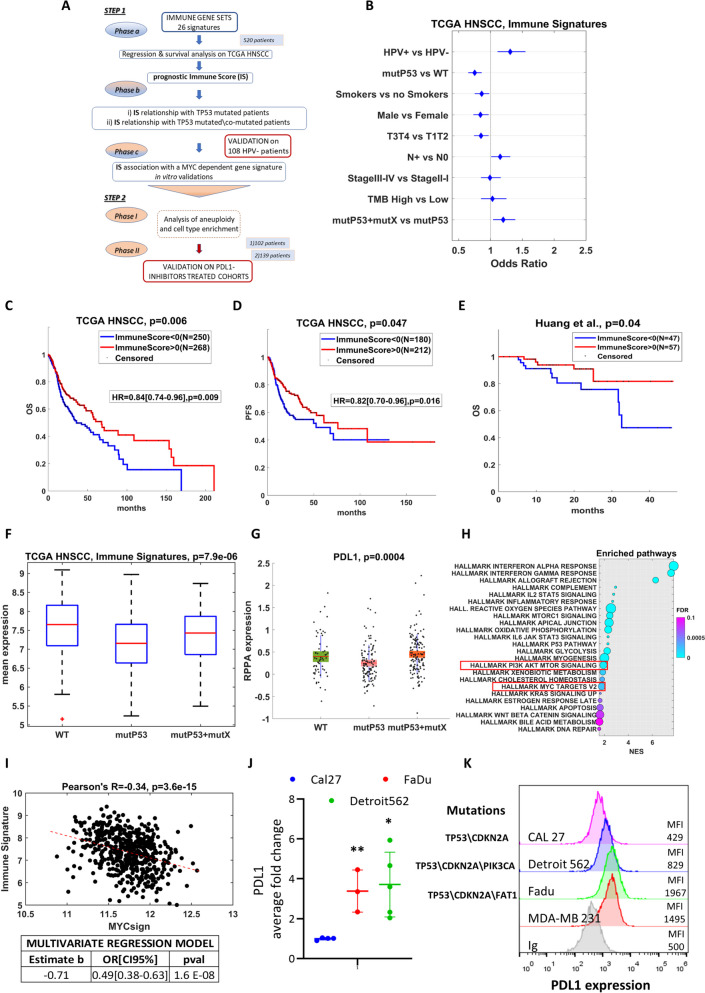
**A** Workflow of the main analyses. **B** Forest plot representing the association of average expression of 125 genes included in the 26 immune gene sets and the clinical variables in 520 HNSCC patients from TCGA. Results of the linear regressions are shown as Odds Ratio with confidence intervals at 95%. **C-D** Kaplan–Meier curves of HNSCC patients from TCGA cohort with high or low Immune Scores evaluated for overall survival and progression free survival (panels C and D, respectively). Differences between curves were evaluated by logrank test. Hazard ratios with 95% confidence intervals were assessed by Cox Hazard regression models. Immune Scores were evaluated as the positive and negative z-scores of the average expression of the 125 genes composing the immune gene sets. **E** Overall Survival in a cohort of 108 HPV-negative HNSCC patients (Huang et al.). Patients were divided based on high and low levels of the Immune Score. Differences between curves were evaluated by log-rank test. **F** Distributions of the gene signature composed by the average expression of 125 genes of the immune gene sets by TP53 mutation and TP53 mutation carried on other mutations among FAT1, CDKN2A and PIK3CA in HNSCC patients (106 WT, 171 TP53 and 189 TP53 + mutX). *P*-values were evaluated by KruskalWallis test. **G** Distributions of the PDL1 protein among different mutational status subgroups from a set of 339 HNSCC patients evaluated by reverse phase protein array (RPPA) in the TCGA cohort. **H** Gene set enrichment analysis of co-mutated patients versus TP53 mutated patients in the TCGA HNSCC cohort. The size of the circles indicates the percentage of genes included in the pathway. Pathways are sorted by False Discovery Rate and normalized enrichment score (NES). The PI3K pathway and the MYC pathway activity were highlighted. For the analysis, we used the GSEA 4.2 software (https://www.gsea-msigdb.org/gsea/index.jsp) run in pre-ranked mode with HALLMARK pathways. **I** Pearson’s correlation between the mean expression of 26 immune gene sets (upper panel) and PD-L1 expression values (bottom panel) with the levels of expression of a 22 genes signature MYC dependent (Ganci et al.) in HNSCC patients from TCGA. A Multivariate regression models were built to adjust the differences of the genes between patients with high and low MYC signature. The models include T status, TP53 mutation, gender, smoking status and, HPV status. High and low expression of the MYC signature were evaluated by positive and negative z-scores of the mean gene expression, respectively. **J** qRT-PCR analysis of PD-L1 in Cal27, FaDu and Detroit 562 cell lines. Statistics (t-test): * *p* < 0.01, ** *p* < 0.005. **K** Flow cytometry analysis of PD-L1 surface expression in cell lines. Representative cell lines (color-coded) were harvested from their cultures and stained with CD274-PE mAb or control Ig for 30 min at 4 °C. Surface expression was assessed on single, live cells on the Attune NxT cytometer. Mean fluorescence intensity is shown. The staggered plot depicts cell line expression according their mutational status

To test the hypothesis that *TP53* gene status and co-driver mutations could be combined to prognostically stratify HNSCC patients and potentially influence the response to ICI, we used a step-by-step approach depicted in the Fig. 1A flowchart.

Step 1 of the flowchart includes the computational analysis of the clinical genomics data from TCGA. Phase (A) describes the analysis of 26 immune gene expression signatures as prognosis predictors within the TCGA cohort of 520 HNSCC patients. A global immune score (IS) for the 26 gene sets considered was obtained as the z-scores of the average expression of the genes included in the gene sets. Phase (B) describes the analysis of the immunosignature prediction performance by HNSCC TCGA subgroups, such as *TP53* wild type (*TP53*-WT) versus mutated (*TP53*-mut), alone (i) and with other co-mutated genes (ii). Phase (C) describes the analysis of association between IS and a 22 MYC-related genes expression. In our previous work we identified a 22-gene MYC-related signature in HNSCC cancer, which is specifically activated by *TP53* mutations with a gain of function activity, and could therefore serve as a proxy for such mutations [10]. In Step 2, we evaluated the impact of aneuploidy in the immune signatures prediction and we performed a cell type enrichment among subgroup of patients with different mutational status. Finally, two validation cohorts of 102 and 139 HNSCC patients treated with PD-L1 inhibitor from the GEO database and MSKCC dataset were analyzed.

### STEP 1 - Phase A: Regression and survival analysis

**Fig. 2:**
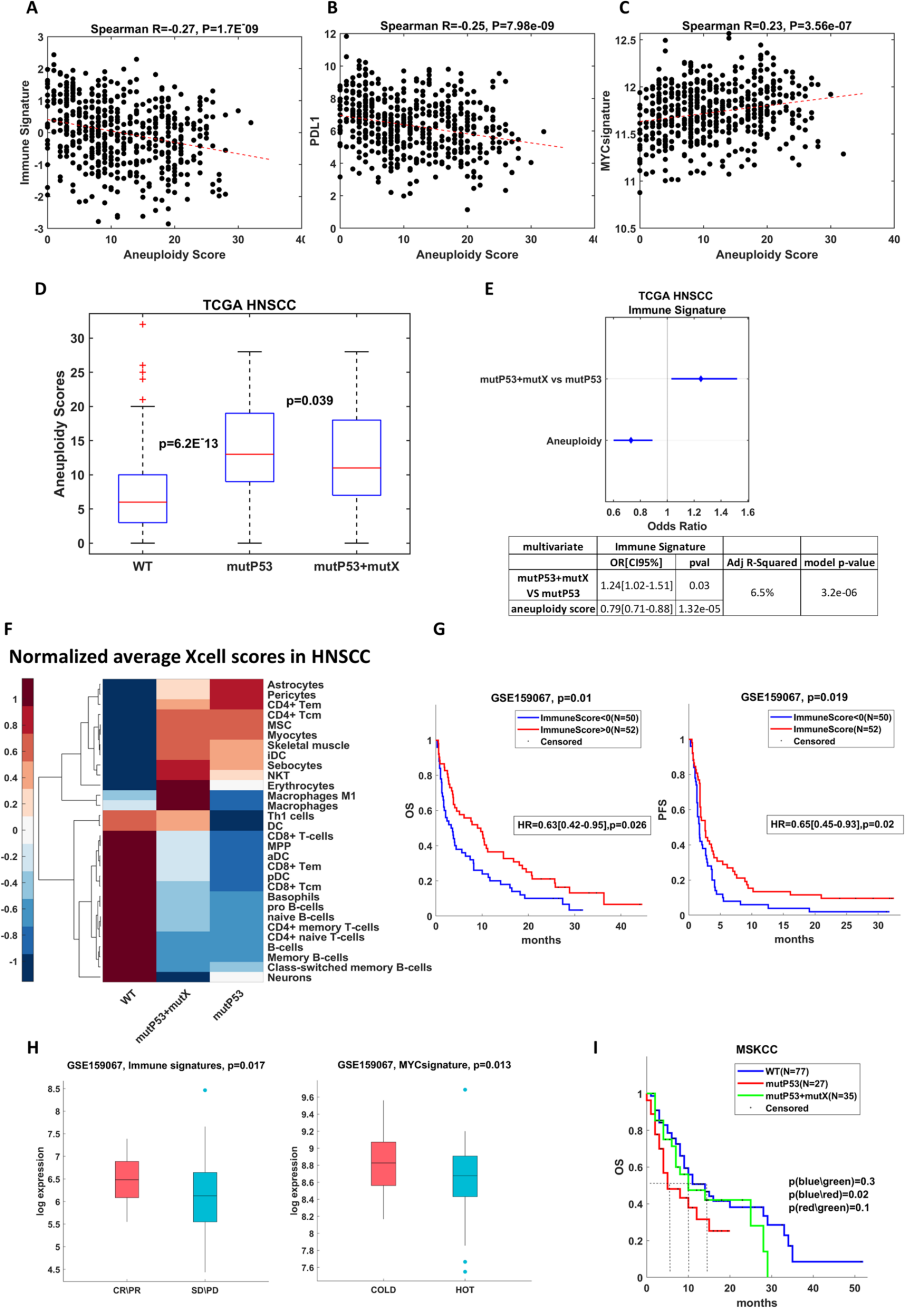
**A** The Spearman’s correlation coefficient reveals a negative association between aneuploidy score and immune signature. **B** Spearman's correlation of PDL1 with aneuploidy scores in TCGA HNSCC patients. **C** Spearman's correlation of the 22-gene MYC signature (Ganci et al.). **D** Distributions of the aneuploidy scores between TP53 mutated patients, WT patients and co-mutated patients. Co-mutated patients show lower aneuploidy than TP53 mutated patients. Statistical significance was evaluated by Wilcoxon test. **E** Forest plot and multivariate regression model to assess the weights in the immune gene sets prediction of the aneuploidy score and the TP53 co-mutational status. The variables resulted to be independent predictors of the immune signature. **F** Cell types enrichment analysis by comparing 64 cell type signatures in subgroups of HNSCC patients with TP53 mutation, TP53 mutation with other mutations and wild type patients. Heatmap representing the normalized average scores obtained from Xcell software, reflecting the cell type abundance of the most significant modulated cell types among the three subgroups. The statistical significance (*p* < 0.05) was assessed by KruskalWallis test. **G** Overall survival (left panel) and Progression free survival (right panel) of 102 patients treated with PDL1 inhibitors from GEO database (GSE159067). Patients were split basing on the Immune Score. The high\low levels of Immune Score were obtained considering the positive and negative z-scores of the average expression of the 26 immune gene sets, respectively. Differences between curves were evaluated by logrank test. The multivariate Cox Hazard regression analysis was adjusted for gender and HPV status. **H** Average expression of the 26 immune gene sets and MYC signature distribution in 102 patients treated with PDL1 inhibitors (GSE159067, left and right panel, respectively). The immune gene sets expression was evaluated in patients with complete or partial response and patients with stable disease or progression disease after treatment (Fig. 2H, left panel). The MYC signature expression was evaluated according to the phenotype classification (“COLD” and “HOT” patients) obtained from Foy JP and colleagues (Fig. 2H, right panel). Differences were evaluated by Wilcoxon test. **I** The overall survival of 139 HNSCC patients in Samstein's cohort (MSKCC) who underwent ICI treatment was analyzed based on their mutational status. *P*-values were assessed using the logrank test

In Fig. 1, panel B, we aimed to identify subgroups of HNSCC patients with a significant difference of IS levels. The first set of results are represented by the forest plot with Odds ratio with 95% CI of demographic and prognostic predictors of the immune signature expression by using regression models in the HNSCC dataset from TCGA. All the analyses were performed at univariate level considering the effect of only one independent variable (demographic or prognostic variables) on the signature (dependent variable). We observed that only the HPV status, with HPV negative versus the positive lesions, lymphnode status (N0 vs N +) and *TP53*-mut plus additional mutations versus *TP53*-mut-only lesions, were statistically associated with a higher immune signature expression. Details of regression analyses on clinical factors for each immune gene set are shown in S-Fig. 1 and S-Fig. 2. Notably, building a multivariable regression model, *TP53* mutational status resulted in the main clinical factor significantly associated with the immune signature (Table S3). While the relationship between *TP53*-mut patients and HPV-negative individuals is established, our observations reveal comparable percentages of HPV-negative patients in *TP53*-mut and *TP53*-mut plus additional mutations groups (91% and 93%, respectively). Taken together, these results suggest that the association between the immune gene set and mutational status, as defined by our categorization, remains unaffected by HPV status. To provide clinical meaning to the described results, in Fig. 1, panel C and D, we reported overall-survival (OS) and progression-free-survival (PFS) curves assessed in the TCGA cohort of 520 patients. In that cohort, high immune signature expression was significantly associated with both overall and progression-free survival. In Fig. 1, panel E, the prognostic value of the immune signature was further validated in the HNSCC CPTAC cohort, as described by Huang et al. [11]. The cohort consisted of 108 HPV-negative patients. This validation study aimed to assess the potential of the immune signature as a prognostic marker specifically for this subset of HNSCC patients confirmed the findings reported for TGCA (Fig. 1E). Additionally, in Supplementary Figure S-Fig. 3, we presented the prognosis of HNSCC patients in the TCGA cohort. Panel A excluded patients with pM1, while panel B excluded HPV-positive patients. This analysis provided insights into the different prognoses observed within the TCGA cohort based on these specific criteria.

### STEP 1 - Phase B: Immunosignature in TP53 mutated (i) and TP53 co-mutated patients (ii)

Because the mutational *TP53* status was so important, we assessed the expression distribution of the immune signature by three groups of patients with *TP53*-WT status, *TP53*-mut status, and *TP53*-mut in combination with one of the other three most frequent mutations observed in HNSCC cancer patients (FAT1, CDKN2A, and PIK3CA genes), hereinafter denoted as *TP53*-mut^+^. *TP53*-WT patients were characterized by a higher IS expression level compared with TP53-mut and *TP53*-mut^+^ patients (Fig. 1F). We considered gene mutation regardless of mutation for statistical power considerations. However, most mutations are missense as described in the S-Fig. 4. Surprisingly, *TP53*-mut^+^ patients had a significantly higher IS score in comparison to the *TP53*-mut patients (Tukey’s post-hoc test, *p* < 0.05) though significantly lower than WT patients (Tukey’s post-hoc test, *p* < 0.01). Congruently, PDL1 protein expression was significantly higher in TP53-WT than in TP53-mut patients. The presence of a co-mutation over that on TP53 exhbited PDL1 protein expression comparable to TP53-WT patients (Fig. 1G). These findings were also found analyzing the HNSCC CPTAC cohort (S-Fig. 5B, C, and D, respectively), thereby providing further robustness to the findings reported for TGCA analysis. To ensure that the differential expression of the immune signature was not influenced by other genomic alterations, we conducted a comparative analysis using copy number alterations instead of co-mutations. Interestingly, we found no significant variation in the immune signature between patients with mutated TP53 and those with TP53 mutations along with other genomic alterations (S-Fig. 5A). This might suggest that the observed differences in the immune signature were specifically associated with *TP53* mutations in presence of other co-mutations (*TP53*-mut^+^).

In Fig. 1H, we performed a gene set enrichment analysis (GSEA) using a ranking list of all genes, comparing their expression between co-mutated and TP53 mutated HNSCC patients. The majority of enriched pathways were related to the immune system, thus reinforcing the association between co-mutations and immune-related processes. Notably, we observed enrichment in MYC-related and PI3K/mTOR pathways among those enriched in the GSEA (Fig. 1H).

In TCGA HNSCC, we observed a negative correlation between the TP53 mutated-dependent MYC signature (from Ganci et al. [10]) and the immune signature (Fig. 1I). Furthermore, in S-Fig. 5E, we found a correlation between the gene PDL1 and the TP53 mutated-dependent MYC signature. These findings suggest the existence of a potential regulatory relationship between TP53, MYC, and immune-related pathways.

In addition, we found that PDL1 mRNA expression is higher in HNSCC cell lines carrying TP53 and additional co-mutations, such as Detroit-562 (TP53\CDKN2A\PIK3CA) and FaDu (TP53\CDKN2A\FAT1) compared to CAL27 carrying TP53\CDKN2A mutations (Fig. 1J). These findings paired with increased PDL1 surface staining in Detroit-562 and FaDu when compared to CAL27 HNSCC cell lines (Fig. 1K).

### STEP 1 - Phase C: Association of a TP53 mutated-dependent MYC signature with the immune gene sets

To further detail the functional link between *TP53* gene mutations with gain of function activity and immune signature, we assessed the role of the *TP53* mutated-dependent MYC signature identified in our previous work [10]. In Fig. 1I, we assessed the correlation of the expression of the immune gene sets and PDL1 (S-Fig. 5E) in TCGA patients with high or low expression of MYC-related signature. A comparison of the same subgroups of patients was also conducted for CTLA4 in TCGA and for the immune gene sets in another HNSCC cohort from GEO (GSE195832). Again, lower expression level of the TP53 mutated-dependent MYC signature was significantly associated with higher levels of the IS score, PDL1 and CTLA4 (S-Fig. 6A, B, C and D). Furthermore, in TCGA cohort we had sufficient clinical information and sample size to adjust those modulations for potential confounding factors. The multivariate models reported at the bottom of panels A, B and C confirmed that those associations, between genes or IS and the MYC-dependent signature, were independent from other clinical factors. Interestingly, c-MYC protein expression was significantly higher in TP53-mut than in TP53-WT patients (S-Fig. 6E). In NCI-60 cell lines, indeed, we found that *WT-TP53* HNSCC cell lines exhibit higher expression of CTLA4 and PDL1 when compared to cell lines harbouring *TP53* mutations (S-Fig. 7A). We also observed that depletion of either mutant p53 protein or its co-factor YAP released PDL1 expression in HNSCC cell lines (S-Fig. 7B).

Enhanced expression of PDL1 was also obtained in CAL-27 and Detroit-562 after treatment with alpelisib, a selective inhibitor of p110α-subunit of PI3K (S-Fig. 7C).

We have previously identified that mutant p53 and YAP proteins favour c-Myc stability and its transcriptional activity in HNSCC cell lines [10]. In that context the use of alpelisib has been found to partially impair this pro tumorigenic axis [10].

To validate these results, we performed qRT-PCR analysis of PD-L1 (S-Fig. 7D) and CTLA4 (S-Fig. 7E) in Cal27 cells, a head and neck cancer cell line carrying a *TP53* mutation, treated with JQ-1. The latter is a small-molecule that inhibits the activity of the BET family proteins by masking their bromodomain acetyl-lysine-binding pockets [12]. JQ-1 has been demonstrated to act as an antineoplastic agent by mainly inhibiting c-MYC functions. Both genes showed increased expression after treatment when compared to their controls, strengthening the potential role of MYC in this immunogenic context.

### STEP 2 - Phase I: Analysis of aneuploidy and cell type enrichment

To study the potential cause of the difference in IS expression between TP53-mut and TP53-mut + HNSCC patients, we considered the aneuploidy score of TCGA HNSCC patients. In general, aneuploidy is strongly associated with TP53 mutations, and is negatively correlated to several immune signatures across various cancers [13]. In line with previous evidence, we observed the negative correlation between aneuploidy scores and IS expression (Fig. 2A) and PDL1 expression (Fig. 2B). Additionally, we observed that higher levels of aneuploidy scores are associated with an increase in the MYC-dependent signature (Fig. 2C). As expected, aneuploidy levels were significantly higher in the TP53-mut and TP53-mut + patients (Fig. 2D). Interestingly, however, aneuploidy levels were significantly lower in the TP53-mut + group relative to the TP53-mut group. Therefore, aneuploidy levels may underlie the difference in IS expression between the two groups. To establish the association between TP53 mutation, co-mutation and aneuploidy levels in immune gene prediction set, we built multivariate regression models, adjusting the TP53 mutational and co-mutational status for the aneuploidy scores. In the multivariate models TP53 co-mutation (*TP53*-mut^+^) and aneuploidy were found to be independent predictors of the immune signature (Fig. 2E). A negative correlation was also found between aneuploidy and CTLA-4 and genes from PI3K\mTOR pathways (S-Fig. 8).

In line with the importance of the TP53 status potentially influencing response to ICI, an analysis of cell type composition, performed with Xcell software [14], revealed distinct immune cell composition across the three TP53 groups (Fig. 2F). In support of a fundamental difference between TP53-mut tumors with vs. without additional mutations, the abundance of 7 immune cell types was statistically different between TP53-mut and TP53mut + patients (S-Fig. 9).

### STEP 2 - Phase II: Analysis of the immune gene sets and MYC dependent signature in two cohorts of HNSCC patients treated with PDL1-inhibitors

We further investigated the role of the immune gene sets and of the TP53 mutated-dependent MYC dependent gene signature in two well characterized cohorts of HNSCC patients under treatment with PD-L1 inhibitors obtained from the GEO database (accession ID: GSE159067) and MSKCC dataset (cBioPortal.org [15],)

In Fig. 2G, we present the results of the analysis regarding the prognostic value of the immune gene sets. The left panel represents overall survival (OS), while the right panel represents progression-free survival (PFS). These results reinforce the findings from both the TCGA cohort and the CPTAC cohort (as shown in Fig. 1C, D, and E), providing further evidence that the expression-based immune signature (IS) score is associated with improved survival outcomes across multiple clinical datasets. The immune gene sets, we used to define our immune score, was also strongly correlated to the classification (“COLD” and “HOT”) introduced by Foy and colleagues (S-Fig. 10A). Notably, in Fig. 2H, left panel, low levels of the immune gene sets are significantly associated with stable or progressive disease during immunotherapy. Furthermore, low level of the *TP53*-dependent MYC signature was significantly associated with the immunologically “HOT” type (Fig. 2H, right panel). Furthermore, we conducted survival analysis on a cohort of 139 patients with Head and Neck cancer who underwent ICI treatment, and whose mutational status was established (MSKCC dataset). Despite the relatively modest sample size of the subgroups, it is apparent that even though the *p*-values may lack robustness, the disparity is striking. Specifically, the median survival among patients with co-mutations is twice that of patients solely harboring the TP53 mutation (10 months versus 5 months, respectively), as illustrated in the Fig. 2I. These results are in line with our finding on TCGA data and cell lines about the potential role of MYC in an immunogenic context. As further evidence supporting the role of co-mutational status, we assessed the expression of the 27-gene set used by Foy and colleagues to define their HOT score in TCGA HNSCC patients with different mutational statuses. Remarkably, we observed that co-mutated patients exhibited a “hotter” profile compared to TP53 mutated patients (S-Fig. 10B).

Finally, we used specific marker genes of the cell types identified in the cell enrichment analysis of TCGA data (Fig. 2F) to evaluate their quantitative expression on immunotherapy treated patients. Six out of the seven investigated cell types resulted strongly up-regulated in HNSCC patients characterized by complete or partial response to the treatment. The same cell types showed a significant reduced abundance in patients with low Immune Score (S-Fig. 10C).

## Discussion

The successful implementation of precision medicine is highly based on clinically-relevant predictive biomarkers. Currently very few markers are known. The administration of ICIs has significantly improved treatment outcomes and survival of HNSCC patients. However, only a limited subset of HNSCC patients benefits from ICI treatment, highlighting the unmet need to better stratify patients for this treatment [16, 17]. In the present work, we used gene expression profiling of a large well-characterized database of HNSCC patients (TCGA), to identify new biomarkers of immune modulation in HNSCC, a disease in which no biomarkers, except for PD-L1 for pembrolizumab, have been identified to date. Of note, while in other tumor types variables such as TMB and tumor stage appear as a noteworthy aspect for the response to ICIs, in HNSCC tumor these variables seem to lose relevance when looking at outcomes adjusted specifically for HPV and TP53 status (Fig. 1B). We additionally discovered associations between variables such as HPV positivity versus negativity, lymph node status, and the *TP53* wild-type (*TP53*-WT) versus *TP53*-mutated (*TP53*-mut) patient groups, which were correlated with elevated levels of both IS as well as increased expression of PDL-1 and CTLA-4. We also found that HNSCC cell lines carrying *TP53*-WT exhibit higher expression levels of both PDL1 and CTLA4 when compared to cell lines bearing *TP53* mutations (S-Fig. 7A). We have previously shown that gain of function activity of *TP53* missense mutations in HNSCC also acts through the aberrant transcriptional activation of a MYC-responsive 22 gene signature that is curtailed by the PI3K inhibitor alpelisib [10]. We found that HNSCC patients with low expression of this MYC signature expressed higher levels of both PDL1 and CTLA4 in comparison to those high levels of the signature. Interestingly, HNSCC patients with low expression of *TP53*-dependent MYC signature have higher immunoscore. Of note, HNSCC patients carrying co-mutations such *TP53/FAT1, TP53/CDKN2A, TP53/PIK3CA* exhibited a higher immunoscore than those with only *TP53* mutations. Consistently, PDL1 expression levels both at transcript and surface staining were higher in HNSCC cell lines carrying *TP53* co-mutations (*TP53*-mut^+^) compared to those carrying only *TP53* mutation (Fig. 1J-K). Depletion of either mutant p53 protein or its co-factor YAP released PDL1 expression in HNSCC cell lines (S-Fig. 7B). The treatment with alpelisib, a selective inhibitor of p110α-subunit of PI3K in CAL-27 and Detroit-562 head and neck cell lines enhanced the expression of PDL1 and CTLA-4 (S-Fig. 7D and E, respectively). To further define differences in immune activity between *TP53*-mut patients and *TP53*-mut + patients, we considered the aneuploidy scores available from cBioPortal (https://www.cbioportal.org/) for TCGA HNSCC cohort. Indeed, the association between *TP53* mutation and aneuploidy has been reported in several human cancers [13]. Herein we originally broaden this association showing that *TP53* mutated patients with higher level of aneuploidy exhibit also lower level of immune gene set expression as described in Fig. 2A and D. High aneuploidy and *TP53* mutational status are both significantly associated to lower immunoscore as shown in the multivariate model (Fig. 2E). Notably, patients harboring a *TP53* mutation in addition to other mutations exhibit a longer median survival of 10 months compared to patients carrying only a *TP53* mutation (5 months), even within a cohort of HNSCC patients treated with ICI (see Fig. 2I). In aggregate, our findings unveil a scenario in which gene mutations, aberrant DNA content and altered gene expression classify more accurately HNSCC patients than each one per se.

## Conclusion

There are few important implications emerging from these findings. Firstly, while *TP53* gain of function mutant p53 proteins might directly repress the expression of ICs such as PDL1, the presence of a co-mutation mitigates this effect through a yet unidentified compensatory mechanism. Given that *TP53*-mut + tumors are less aneuploid than *TP53*-mut-only tumors, and that high degree of aneuploidy is associated with escape from immune-surveillance [13, 18], aneuploidy might contribute to the drug response differences between the groups. Secondly, HNSCC patients carrying co-mutations TP53/PIK3CA could benefit from alpelisib plus ICI. Thirdly, HNSCC patients relapsing to the PI3K inhibitor alpelisib might be proposed for immunotherapy treatment. It should be acknowledged that PI3K inhibitors have been studied [19] and are currently in clinical trials also in RM-HNSCC (NCT04338399). With the due limitations, deconvolution analyses from bulk-RNA-Seq data revealed that high/low immunoscore might contribute to deciphering the immune infiltration cellular landscape of HNSCC patients. Indeed, we found that high-immunoscore HNSCC patients exhibited immune infiltration in which aDC, macrophages, CD8 + T-, macrophages M1, naïve B cells, pDC, Th1 cells appear to be significantly more represented than in those with low immunoscore. Notably, HNSCC patients with TP53 and co-mutations showed a putative immune cellular landscape more similar to *TP53*-WT HNSCC patients than to those with TP53 mutations. There are no current therapies that directly promote infiltration of immune cells for HNSCC patients, but the potential of the identified immunoscore to provide insights into the cellular composition of the immune infiltrate is certainly relevant to profile immunologically a given patient. Therefore, our findings provide evidence of an immunoscore that holds prognostic features of a biomarker which contributes to accurately classify HNSCC patients.

### Supplementary Information

**Additional file 1:** **Fig. S1.** A-D) Forest plot representing Odds ratio with 95% CI of clinical predictors of several immune cell types and functional gene sets by using regression models in HNSCC dataset from TCGA. Red line highlights the behaviour of PD-L1 for comparison with other gene sets. All lines that don’t cross the 1 value are statistically significant. Each variable was dichotomized in the models to compare subgroup of patients by HPV status (A), tumor mutational burden (TMB) (B), and TP53 mutational status in concomitance or not with other mutations among FAT1, CDKN2A, PIK3CA (mutX) (b and c, respectively). **Fig. S2.** A-F) Forest plot representing Odds ratio with 95% CI of clinical predictors of 26 immune cell types and functional gene sets by using regression models in HNSCC dataset from TCGA. Red line highlights the behaviour of PD-L1 for comparison with other gene sets. All lines that don’t cross the 1 value are statistically significant. Each variable was dichotomized in the models to compare subgroup of patients by gender (A), smoking history (B), tumor size (C), lympho-node status (D) and stage (E). **Fig. S3.** A) Overall Survival (left panel) and Disease Free Survival (right panel) in a TCGA cohort of HNSCC patients who did not receive neoadjuvant therapy. pM1 samples were excluded. Patients were divided based on high and low levels of the Immune Signature, defined as positive and negative z-scores of the average expression of immune gene sets, respectively. The Cox hazard regression model was adjusted for gender, TP53 mutation, HPV status, and smoking history. Differences between curves were evaluated by log-rank test. B) Overall Survival in a TCGA cohort of HPV-negative HNSCC patients, divided based on high and low levels of the Immune Score. Multivariate Cox regression was adjusted for gender, TP53 mutation, HPV status, and smoking history. Differences between curves were evaluated by log-rank test. **Fig. S4.** We assessed the proportions of mutation types within the evaluated genes using the TCGA HNSCC cohort, with data sourced from the CbioPortal. The final row displays the total number of mutations considered for the four genes and their respective distribution among mutation subtypes. **Fig. S5.** A) The average expression distributions of the Immune Signature were analyzed based on the mutational status and copy number alterations of three frequently mutated genes in TCGA HNSCC (Alt_X), namely CDKN2A, PIK3CA, and FAT1. Among the patients, genomic alterations were observed in CDKN2A, PIK3CA, and FAT1 genes in 32%, 21%, and 8% of cases, respectively. To evaluate the statistical significance, the Kruskal-Wallis test and Wilcoxon test were employed. B-C) Distributions of the average expression of the Immune Signature (panel B) and PDL1 (panel C) based on the mutational status of 108 HNSCC patients from the Huang et al. cohort. Statistical significance was assessed by the Wilcoxon test. D) Distributions of the average expression of the MYC signature from Ganci et al., based on the mutational status of 108 HNSCC patients in the Huang et al. cohort. Statistical significance was assessed by the Wilcoxon test. E) Spearman's correlation of the 22-gene MYC signature (Ganci et al.) and PDL1 expression. **Fig. S6.** A-C) box-plot of IS (A), PDL1 (B) and CTLA4 (C) expression in patients with high and low level expression of a 22 genes signature MYC dependent (Ganci et al.) in HNSCC datasets from TCGA. Statistical significance between distributions was assessed by Wilcoxon rank-sum test. Multivariate regression models were built to adjust the differences of the genes between patients with high and low MYC signature. The models include T status, TP53 mutation, gender, smoking status and, HPV status. High and low expression of the MYC signature were evaluated by positive and negative z-scores of the mean gene expression, respectively. D) box-plot of the mean expression of 26 immune gene sets in 28 pre-treated HNSCC patients with high and low level expression of a 22 genes signature MYC dependent in GSE195832 dataset from GEO. Statistical significance between distributions was assessed by Wilcoxon rank-sum test. E) Distributions of the c-Myc protein among different mutational status subgroups from a set of 339 HNSCC patients evaluated by reverse phase protein array (RPPA) in the TCGA cohort. *P*-values were evaluated by KruskalWallis test. **Fig. S7.** A) Heatmap of PDL1 and CTLA4 expression from 33 HNSCC cell lines harbouring TP53 mutation and 3 WT cell lines obtained from Iorio et al (array express, E-MTAB-3610) dataset (left panel), and the relative box-plots of the distributions (right panel). Differences were evaluated by Wilcoxon test. B) qRT-PCR analysis of PD-L1 in Cal27 and Detroit 562 cell lines depleted or not of p53 and YAP. Statistics (t-test): * *p* < 0.01, ** *p*<0.005. C) qRT-PCR analysis of PD-L1 in Cal27 and Detroit 562 cell lines treated with 5nM of Byl-719. Statistics (t-test): * *p* < 0.01, ** *p*<0.005. D) qRT-PCR analysis of PD-L1 in Cal27 treated with JQ-1. Bars indicate the average of at least three independent experiments. Statistics (t-test): * *p* < 0.01, ** *p*<0.005. E) qRT-PCR analysis of CTLA4 in Cal27 treated with JQ-1. Bars indicate the average of at least three independent experiments. Statistics (t-test): * *p* < 0.01, ** *p*<0.005. **Fig. S8.** A) Spearman's correlation of CTLA4 with aneuploidy scores in TCGA HNSCC patients. B) Spearman's correlation of the 22-gene MYC signature (Ganci et al.) and the average expression of the genes included in the HALLMARK PI3K_AKT_MTOR_SIGNALING pathway, which are specifically modulated between co-mutated patients and TP53 mutated patients. **Fig. S9.** A) Cell types enrichment in TCGA HNSCC of TP53 mutated patients and TP53 mutated patients who harboured other mutations. Scores were obtained from Xcell software. *P*-values were evaluated by Wilcoxon ranksum test. **Fig. S10.** A) Average expression of the 26 immune gene sets distribution in 102 patients treated with PDL1 inhibitors (GSE159067). The immune gene sets expression was evaluated splitting the patients according to the phenotype classification (“COLD” and “HOT” patients) obtained from Foy JP and colleagues. Differences were evaluated by Wilcoxon test. B) Distribution of the 27-gene signature used by Foy et al. to define the HOT score. The gene set was evaluated in the TCGA HNSCC cohort among WT patients, co-mutated patients, and TP53 only mutated patients. Statistical significance among groups was assessed by the Kruskal-Wallis test.C) Significantly modulated cell type marker genes in 102 patients from GEO database (GSE159067) among the 7 cell types previously identified in the deconvolution analysis of TCGA HNSCC data. Statistical significance between patients with high and low Immune Score was evaluated by Wilcoxon test. Immune Score was defined as z-score of the average expression of the 26 immune gene sets.

**Additional file 2:** **Table S1.** List of the immune gene sets analysed. This table comprises the immune gene sets analyzed, listing all genes utilized in determining the immune score. The immune score was evaluated through the z-score transformation of the average expression derived from the complete gene list. The genes and their respective correlation with immune activity were obtained from Lyu HY et al (Computational and Structural Biotechnology Journal, 2019). **Table S2.** Descriptive characteristics of TCGA HNSCC dataset. **Table S3. **Multivariate regression models of the main clinical variables associated with the immune signatures in HNSCC. The model was built considering a gene signature including all the 125 genes composing the immune gene sets.

## Data Availability

Data derived from the “The Cancer Genome Atlas” (TCGA—the HNSCC-TCGA, Nature 2015) and the analyses included 520 HNSCC patients. We gathered the normalized TCGA HNSCC gene expression of tumour from Broad Institute TCGA Genome Data Analysis Center (http://gdac.broadinstitute.org/): Firehose stddata__2016_01_28 and Broad Institute of MIT and Harvard. https://doi.org/10.7908/C11G0KM9. A validation cohort of 102 HNSCC patients treated with PDL1 inhibitors was gathered from GEO database with accession ID GSE159067. A second cohort of 108 HNSCC HPV negative patients (Huang et al., PMID: 33,417,831) was obtained from CPTAC (https://www.linkedomics.org/login.php#dataSource). A separate validation cohort, comprising mutational status and survival data for 139 HNSCC patients treated with ICI, was obtained from cbioportal.org (Samstein et al., PMID: 30,643,254). Transcriptomic landscape of HN cell lines was obtained from Iorio et al. (array express, E-MTAB-3610, PMID: 27,397,505).

## Abbreviations

R-M: Recurrent/metastatic
FDA: Food and drug administration
PD-1: The anti-programmed cell death protein
ICIs: Immune checkpoint inhibitors
HNSCC: Head and neck squamous cell carcinoma
PDL-1: The anti-programmed cell death protein ligand -1
TMB: Tumour mutational burden
CPS: Combined positive score
CTLA-4: T-lymphocyte associated protein 4
TCGA: The cancer genome atlas
OS: Overall survival
PFS: Progression free survival
aDC: Activated dendritic cell
pDC: Plasmacytoid dendritic cell
