# Supplementary material for: Immunosignatures associated with TP53 status and co-mutations classify prognostically head and neck cancer patients
Source: Mol Cancer. 2023 Nov 30;22:192. doi: 10.1186/s12943-023-01905-9 (PMC10687972; doi:10.1186/s12943-023-01905-9)
Supplement: Supplementary file 1 — Additional file 1: Fig. S1. A-D) Forest plot representing Odds ratio with 95% CI of clinical predictors of several immune cell types and functional gene sets by using regression models in HNSCC dataset from TCGA. Red line highlights the behaviour of PD-L1 for comparison with other gene sets. All lines that don’t cross the 1 value are statistically significant. Each variable was dichotomized in the models to compare subgroup of patients by HPV status (A), tumor mutational burden (TMB) (B), and TP53 mutational status in concomitance or not with other mutations among FAT1, CDKN2A, PIK3CA (mutX) (b and c, respectively). Fig. S2. A-F) Forest plot representing Odds ratio with 95% CI of clinical predictors of 26 immune cell types and functional gene sets by using regression models in HNSCC dataset from TCGA. Red line highlights the behaviour of PD-L1 for comparison with other gene sets. All lines that don’t cross the 1 value are statistically significant. Each variable was dichotomized in the models to compare subgroup of patients by gender (A), smoking history (B), tumor size (C), lympho-node status (D) and stage (E). Fig. S3. A) Overall Survival (left panel) and Disease Free Survival (right panel) in a TCGA cohort of HNSCC patients who did not receive neoadjuvant therapy. pM1 samples were excluded. Patients were divided based on high and low levels of the Immune Signature, defined as positive and negative z-scores of the average expression of immune gene sets, respectively. The Cox hazard regression model was adjusted for gender, TP53 mutation, HPV status, and smoking history. Differences between curves were evaluated by log-rank test. B) Overall Survival in a TCGA cohort of HPV-negative HNSCC patients, divided based on high and low levels of the Immune Score. Multivariate Cox regression was adjusted for gender, TP53 mutation, HPV status, and smoking history. Differences between curves were evaluated by log-rank test. Fig. S4. We assessed the proportions of mutation type [file 12943_2023_1905_MOESM1_ESM.pdf]

Figure S1

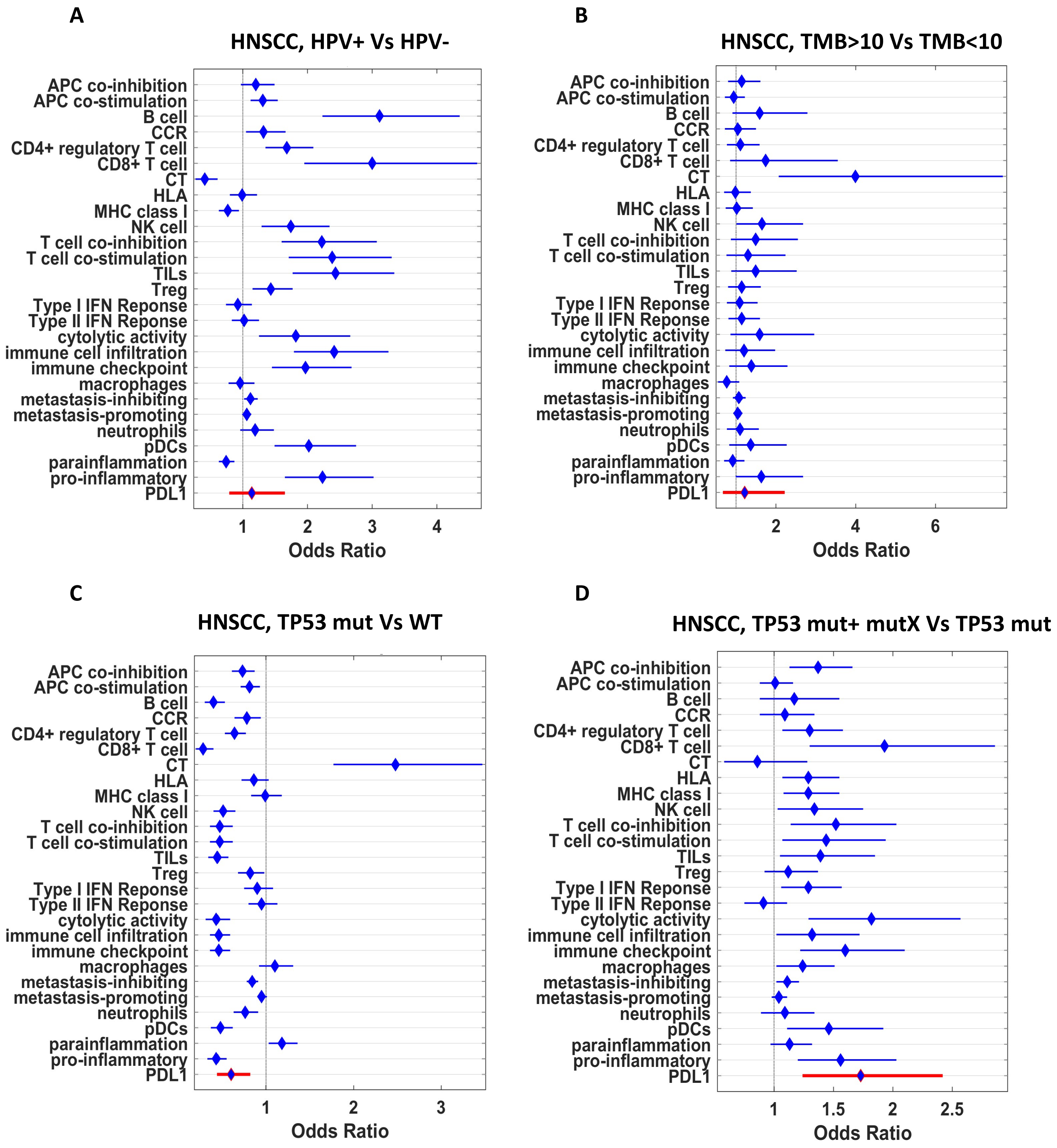
A
HNSCC, HPV+ Vs HPV-
APC co-inhibition
APC co-stimulation
B cell
CCR
CD4+ regulatory T cell
CD8+ T cell
CT
HLA
MHC class I
NK cell
T cell co-inhibition
T cell co-stimulation
TILs
Treg
Type I IFN Reponse
Type II IFN Reponse
cytolytic activity
immune cell infiltration
immune checkpoint
macrophages
metastasis-inhibiting
metastasis-promoting
neutrophils
pDCs
parainflammation
pro-inflammatory
PDL1
1 2 3 4
Odds Ratio
B
HNSCC, TMB>10 Vs TMB<10
APC co-inhibition
APC co-stimulation
B cell
CCR
CD4+ regulatory T cell
CD8+ T cell
CT
HLA
MHC class I
NK cell
T cell co-inhibition
T cell co-stimulation
TILs
Treg
Type I IFN Reponse
Type II IFN Reponse
cytolytic activity
immune cell infiltration
immune checkpoint
macrophages
metastasis-inhibiting
metastasis-promoting
neutrophils
pDCs
parainflammation
pro-inflammatory
PDL1
2 4 6
Odds Ratio
C
HNSCC, TP53 mut Vs WT
APC co-inhibition
APC co-stimulation
B cell
CCR
CD4+ regulatory T cell
CD8+ T cell
CT
HLA
MHC class I
NK cell
T cell co-inhibition
T cell co-stimulation
TILs
Treg
Type I IFN Reponse
Type II IFN Reponse
cytolytic activity
immune cell infiltration
immune checkpoint
macrophages
metastasis-inhibiting
metastasis-promoting
neutrophils
pDCs
parainflammation
pro-inflammatory
PDL1
1 2 3
Odds Ratio
D
HNSCC, TP53 mut+ mutX Vs TP53 mut
APC co-inhibition
APC co-stimulation
B cell
CCR
CD4+ regulatory T cell
CD8+ T cell
CT
HLA
MHC class I
NK cell
T cell co-inhibition
T cell co-stimulation
TILs
Treg
Type I IFN Reponse
Type II IFN Reponse
cytolytic activity
immune cell infiltration
immune checkpoint
macrophages
metastasis-inhibiting
metastasis-promoting
neutrophils
pDCs
parainflammation
pro-inflammatory
PDL1
1 1.5 2 2.5
Odds Ratio

Figure S2

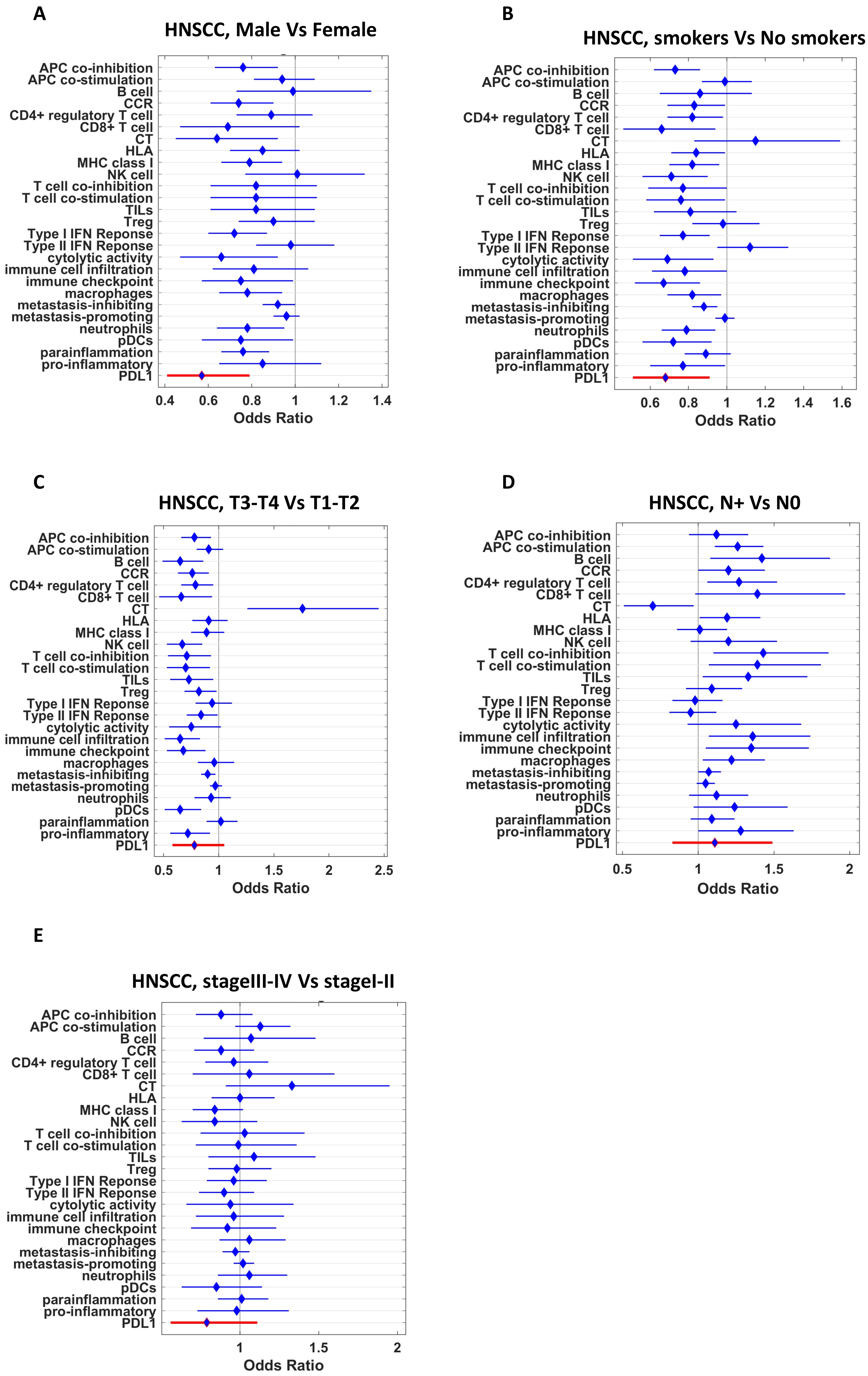
A
HNSCC, Male Vs Female
APC co-inhibition
APC co-stimulation
B cell
CCR
CD4+ regulatory T cell
CD8+ T cell
CT
HLA
MHC class I
NK cell
T cell co-inhibition
T cell co-stimulation
TILs
Treg
Type I IFN Reponse
Type II IFN Reponse
cytolytic activity
immune cell infiltration
immune checkpoint
macrophages
metastasis-inhibiting
metastasis-promoting
neutrophils
pDCs
parainflammation
pro-inflammatory
PDL1
0.4 0.6 0.8 1 1.2 1.4
Odds Ratio
B
HNSCC, smokers Vs No smokers
APC co-inhibition
APC co-stimulation
B cell
CCR
CD4+ regulatory T cell
CD8+ T cell
CT
HLA
MHC class I
NK cell
T cell co-inhibition
T cell co-stimulation
TILs
Treg
Type I IFN Reponse
Type II IFN Reponse
cytolytic activity
immune cell infiltration
immune checkpoint
macrophages
metastasis-inhibiting
metastasis-promoting
neutrophils
pDCs
parainflammation
pro-inflammatory
PDL1
0.6 0.8 1 1.2 1.4 1.6
Odds Ratio
C
HNSCC, T3-T4 Vs T1-T2
APC co-inhibition
APC co-stimulation
B cell
CCR
CD4+ regulatory T cell
CD8+ T cell
CT
HLA
MHC class I
NK cell
T cell co-inhibition
T cell co-stimulation
TILs
Treg
Type I IFN Reponse
Type II IFN Reponse
cytolytic activity
immune cell infiltration
immune checkpoint
macrophages
metastasis-inhibiting
metastasis-promoting
neutrophils
pDCs
parainflammation
pro-inflammatory
PDL1
0.5 1 1.5 2 2.5
Odds Ratio
D
HNSCC, N+ Vs N0
APC co-inhibition
APC co-stimulation
B cell
CCR
CD4+ regulatory T cell
CD8+ T cell
CT
HLA
MHC class I
NK cell
T cell co-inhibition
T cell co-stimulation
TILs
Treg
Type I IFN Reponse
Type II IFN Reponse
cytolytic activity
immune cell infiltration
immune checkpoint
macrophages
metastasis-inhibiting
metastasis-promoting
neutrophils
pDCs
parainflammation
pro-inflammatory
PDL1
0.5 1 1.5 2
Odds Ratio
E
HNSCC, stageIII-IV Vs stageI-II
APC co-inhibition
APC co-stimulation
B cell
CCR
CD4+ regulatory T cell
CD8+ T cell
CT
HLA
MHC class I
NK cell
T cell co-inhibition
T cell co-stimulation
TILs
Treg
Type I IFN Reponse
Type II IFN Reponse
cytolytic activity
immune cell infiltration
immune checkpoint
macrophages
metastasis-inhibiting
metastasis-promoting
neutrophils
pDCs
parainflammation
pro-inflammatory
PDL1
1 1.5 2
Odds Ratio

Figure S3

A

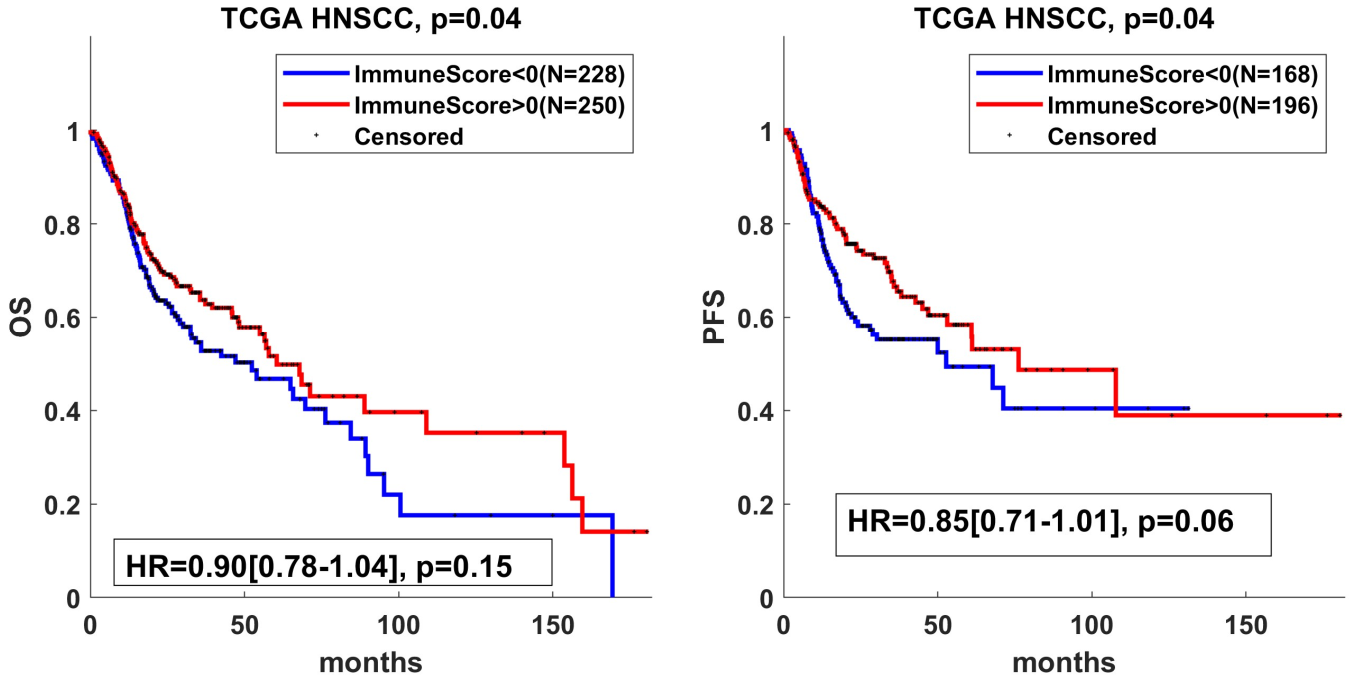
TCGA HNSCC, p=0.04
ImmuneScore<0(N=228)
ImmuneScore>0(N=250)
Censored
OS
HR=0.90[0.78-1.04], p=0.15
months
TCGA HNSCC, p=0.04
ImmuneScore<0(N=168)
ImmuneScore>0(N=196)
Censored
PFS
HR=0.85[0.71-1.01], p=0.06
months

B

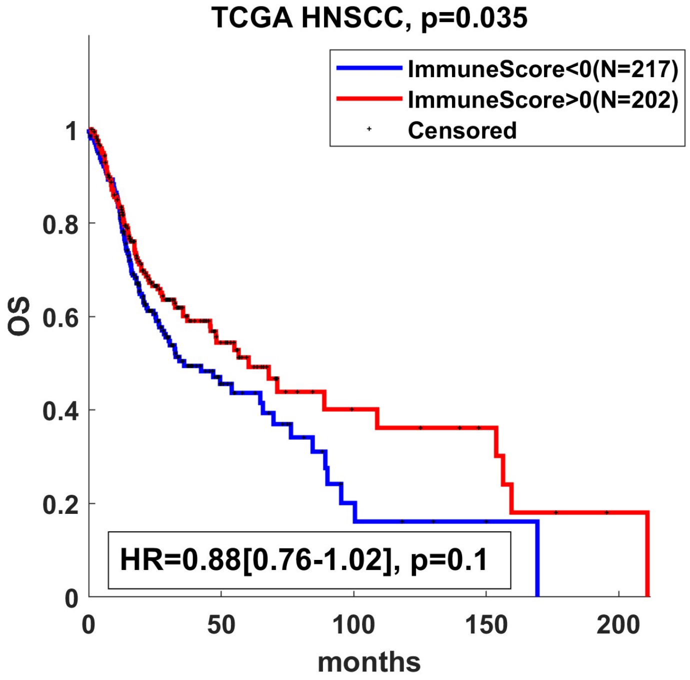
TCGA HNSCC, p=0.035
ImmuneScore<0(N=217)
ImmuneScore>0(N=202)
Censored
OS
HR=0.88[0.76-1.02], p=0.1
months

Figure S4

GENE	MISSENSE	TRUNCATING	SPLICE	INFRAME
**TP53**	53%	20%	13%	14%
**CDKN2A**	18%	52%	13%	17%
**FAT1**	18%	47%	4%	31%
**PIK3CA**	98%	0%	1%	1%
**# total mutations (678)**	48%	27%	10%	15%

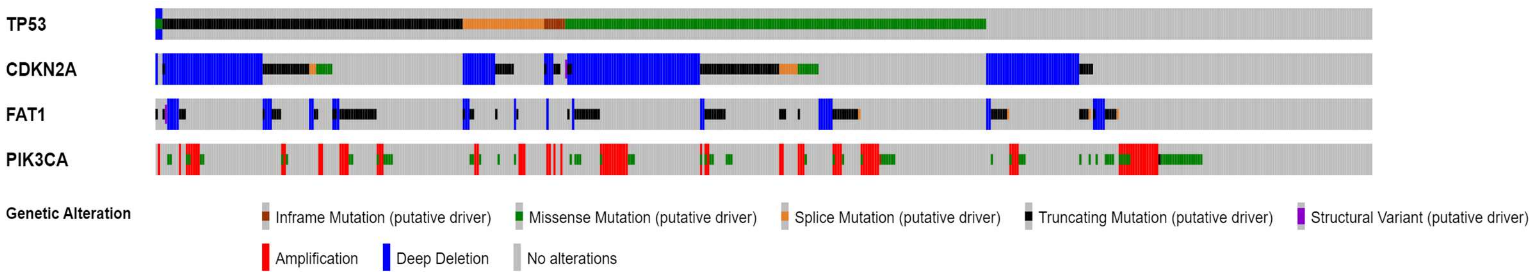

Figure S5

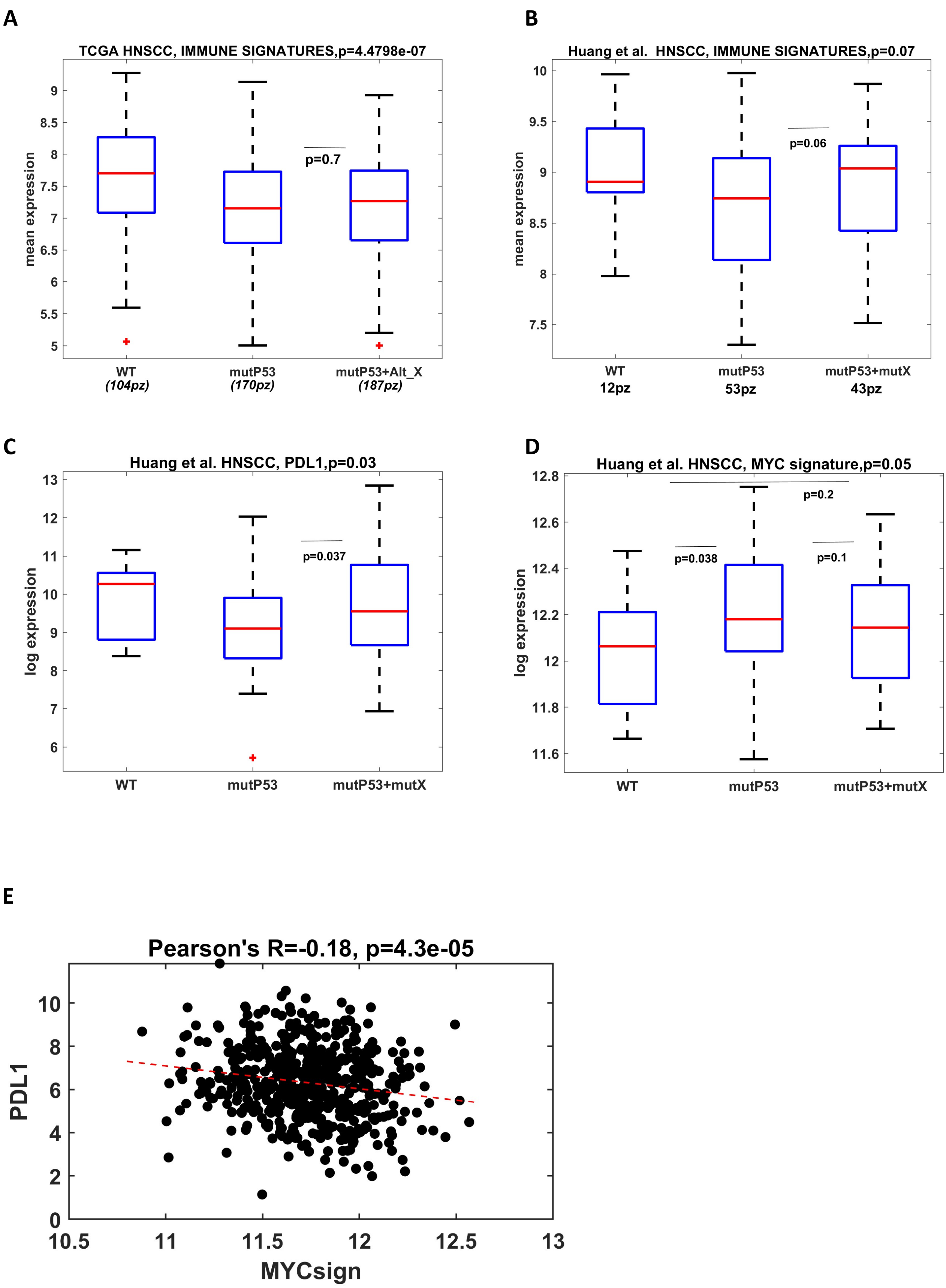

MULTIVARIATE REGRESSION MODEL		
Estimate b	OR[CI95%]	pval
-0.14	0.86[0.77-0.97]	0.01

Figure S6

A

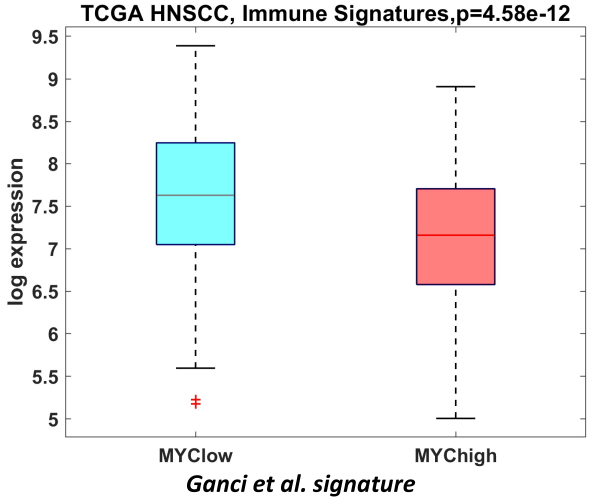

MULTIVARIATE REGRESSION MODEL		
Estimate b	OR[CI95%]	pval
-0.71	0.49[0.38-0.63]	1.6 E-08

B

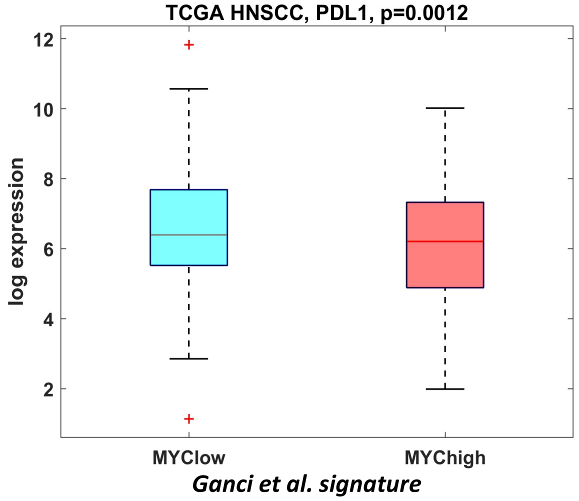

MULTIVARIATE REGRESSION MODEL		
Estimate b	OR[CI95%]	pval
-0.14	0.86[0.77-0.97]	0.01

C

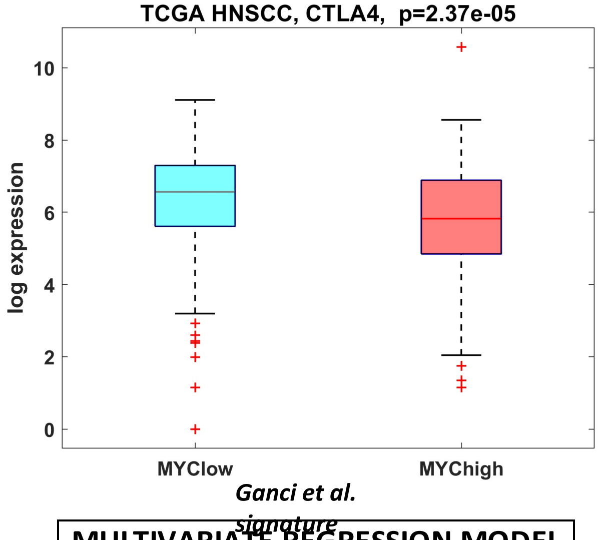

MULTIVARIATE REGRESSION MODEL		
Estimate b	OR[CI95%]	pval
-0.20	0.82[0.72-0.93]	2.1 E-03

D

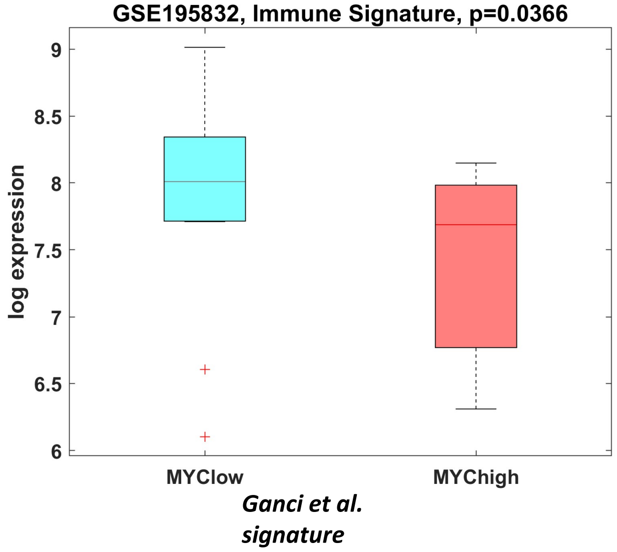

E

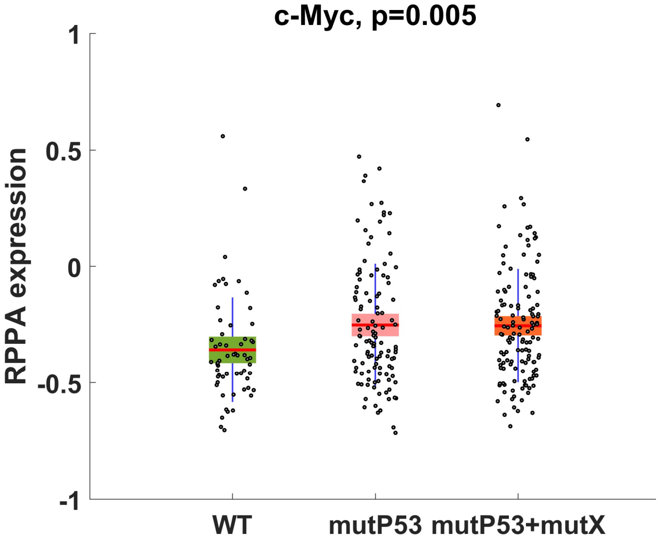

Figure S7

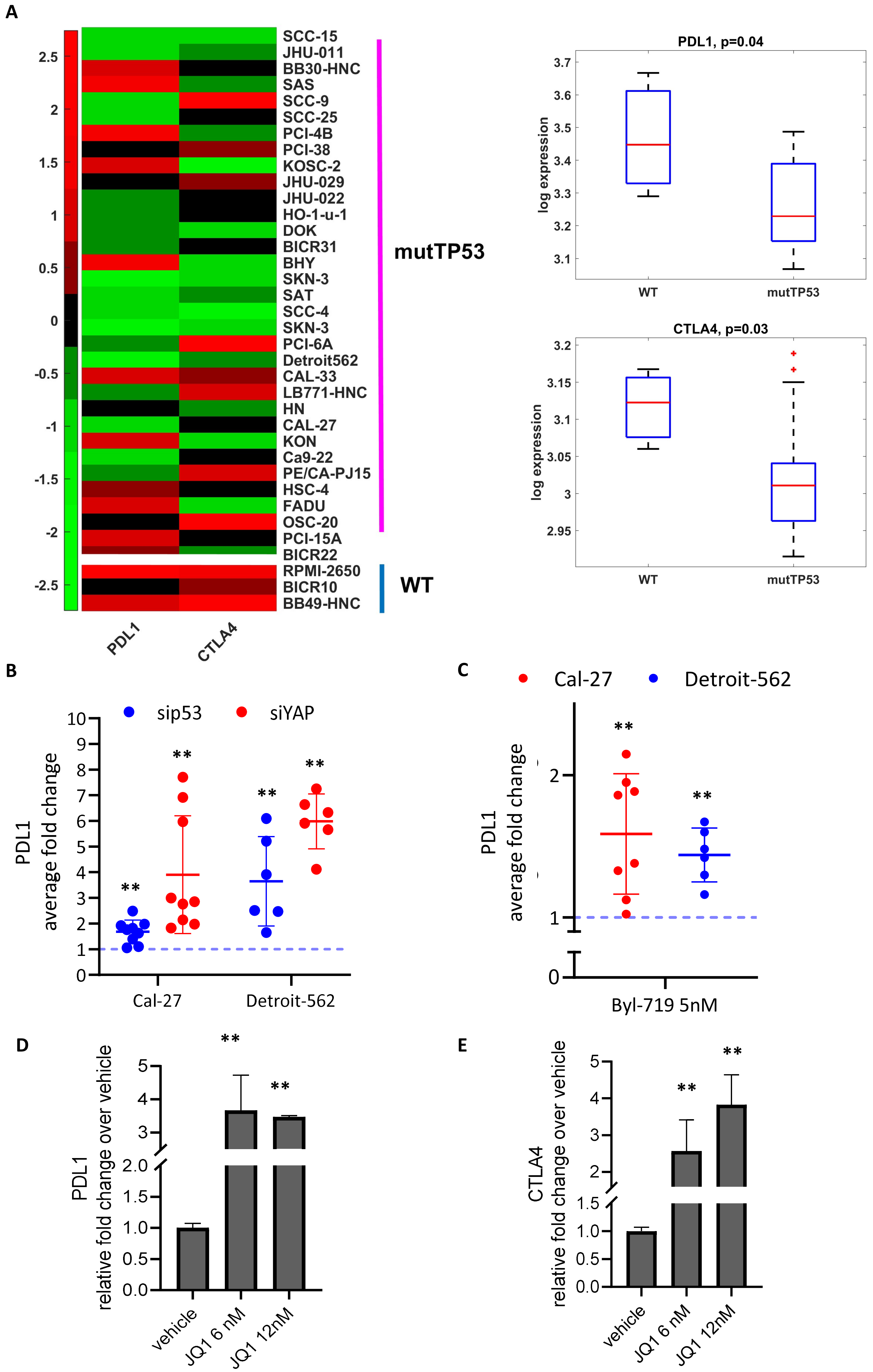
A
2.5
2
1.5
1
0.5
0
-0.5
-1
-1.5
-2
-2.5
SCC-15
JHU-011
BB30-HNC
SAS
SCC-9
SCC-25
PCI-4B
PCI-38
KOSC-2
JHU-029
JHU-022
HO-1-u-1
DOK
BICR31
BHY
SKN-3
SAT
SCC-4
SKN-3
PCI-6A
Detroit562
CAL-33
LB771-HNC
HN
CAL-27
KON
Ca9-22
PE/CA-PJ15
HSC-4
FADU
OSC-20
PCI-15A
BICR22
RPMI-2650
BICR10
BB49-HNC
mutTP53
WT
PDL1
CTLA4
PDL1, p=0.04
log expression
WT
mutTP53
CTLA4, p=0.03
log expression
WT
mutTP53
B
sip53
siYAP
PDL1
average fold change
Cal-27
Detroit-562
C
Cal-27
Detroit-562
PDL1
average fold change
Byl-719 5nM
D
PDL1
relative fold change over vehicle
vehicle
JQ1 6 nM
JQ1 12nM
E
CTLA4
relative fold change over vehicle
vehicle
JQ1 6 nM
JQ1 12nM

Figure S8

A

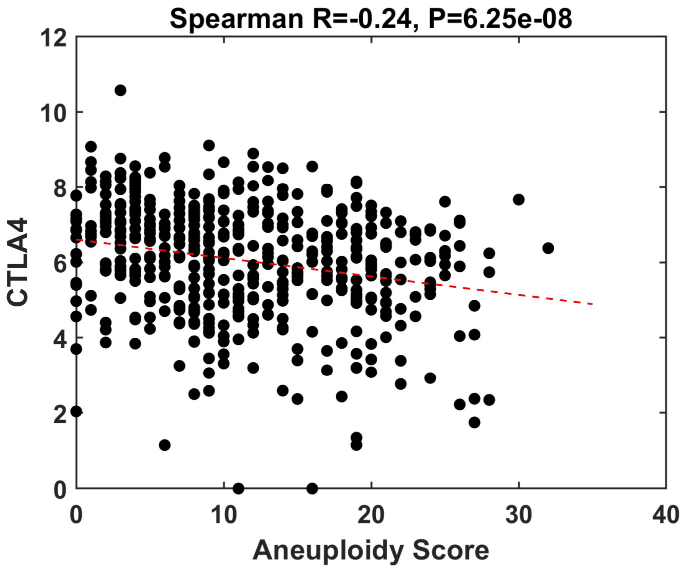
Spearman R=-0.24, P=6.25e-08
CTLA4
12
10
8
6
4
2
0
0
10
20
30
40
Aneuploidy Score

B

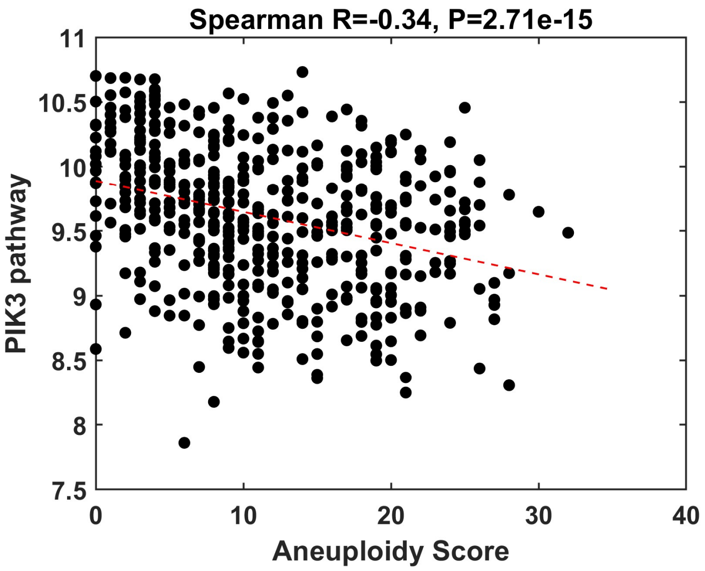
Spearman R=-0.34, P=2.71e-15
PIK3 pathway
11
10.5
10
9.5
9
8.5
8
7.5
0
10
20
30
40
Aneuploidy Score

Figure S9

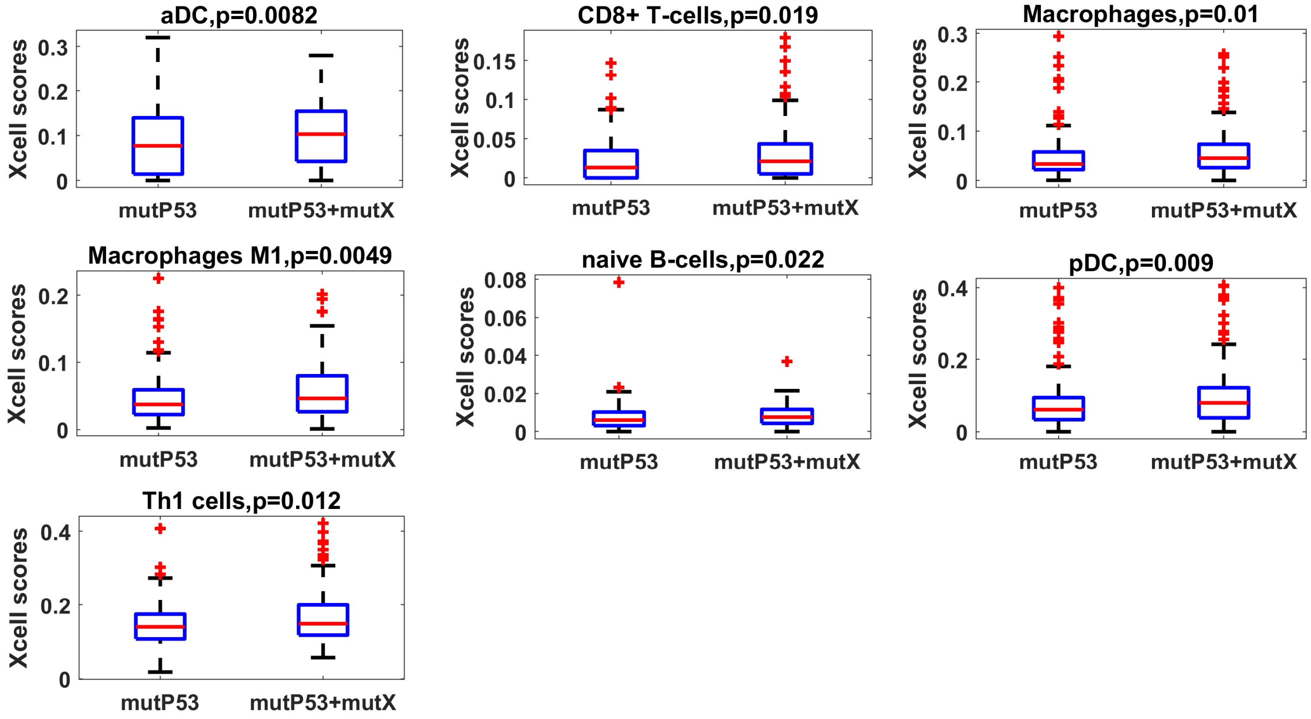
aDC,p=0.0082
CD8+ T-cells,p=0.019
Macrophages,p=0.01
Macrophages M1,p=0.0049
naive B-cells,p=0.022
pDC,p=0.009
Th1 cells,p=0.012
Xcell scores
mutP53
mutP53+mutX

Figure S10

A

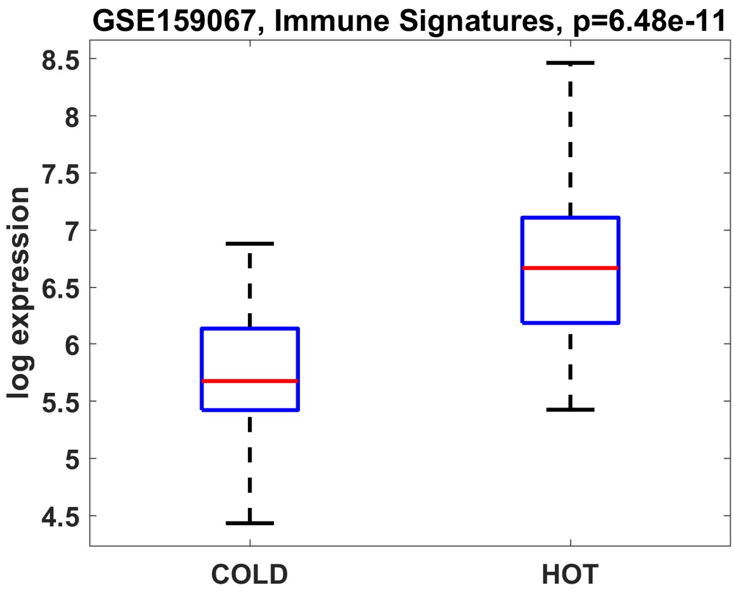

B

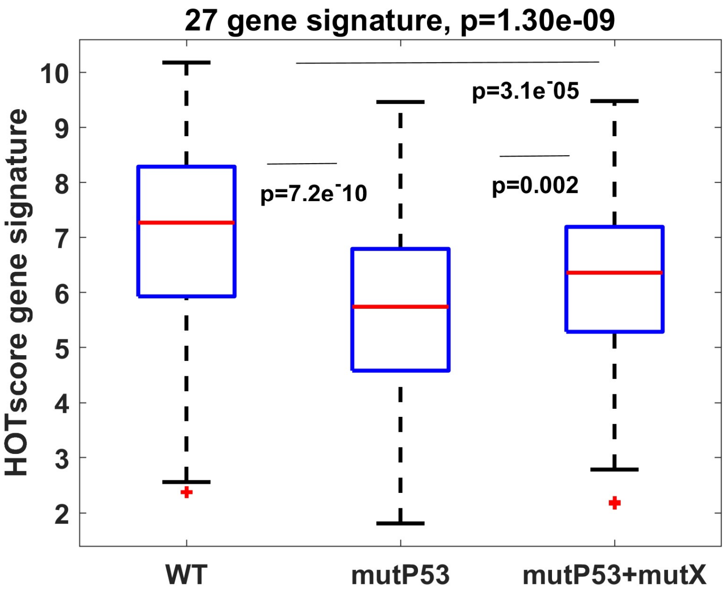

C

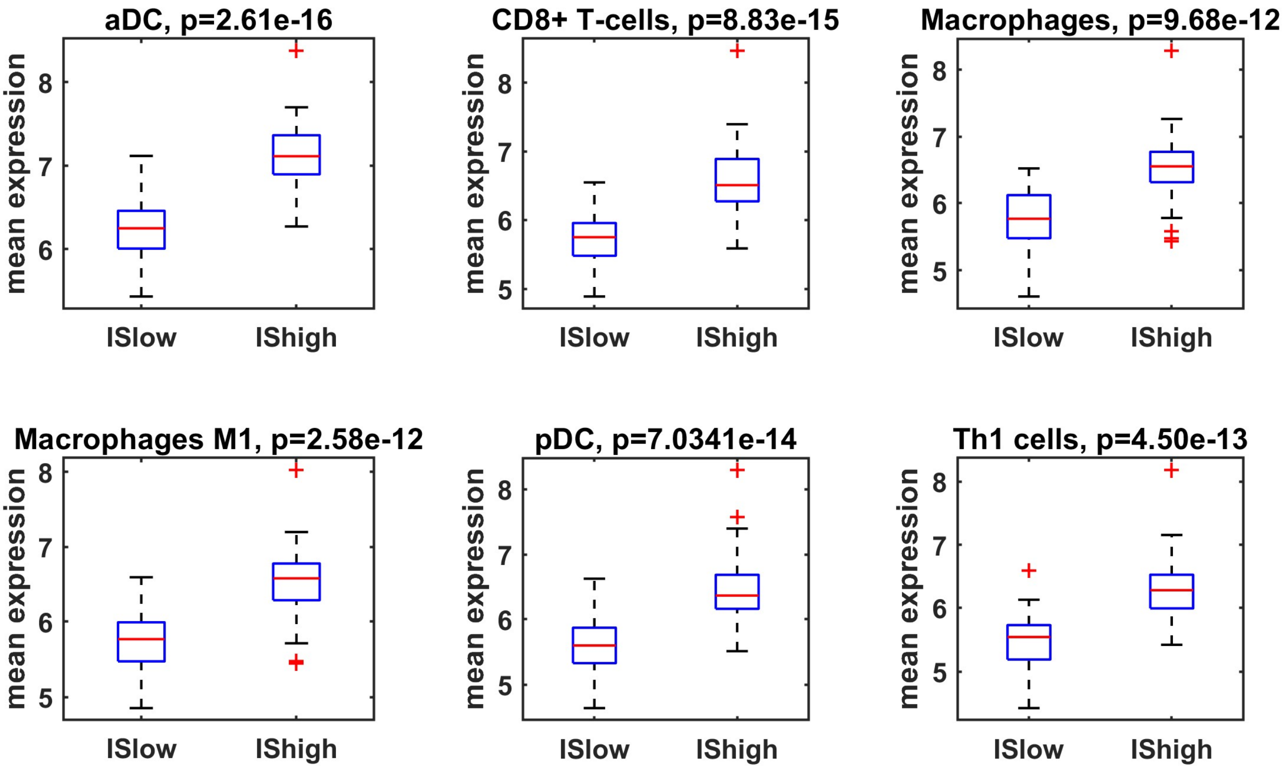

Table S1

	signature	genes
15 immune cell types and function	B cell	CD79B,BTLA,FCRL3,BANK1
	CD4+ regulatory T cell	C15orf53,IL32,CTLA4,FOXP3
	CD8+ T cell	CD8A
	NK cell	KLRF1,KLRC1
	cytolytic activity	GZMA,PRF1
	macrophages	CD68,CYBB,MMP9,LGMN
	MHC class I	HLA-A,B2M,TAP1
	APC co-stimulation	ICOSLG,CD70,CD40,CD58
	T cell co-stimulation	CD27,CD28,ICOS,CD2,CD226
	APC co-inhibition	CD274,PDCD1LG2,C10orf54,LGALS9
	T cell co-inhibition	CTLA4,LAG3,TIGIT,BTLA
	neutrophils	SELL,VNN3,KDM6B,MNDA
	pDCs	IRF8,GZMB,CXCR3,CLEC4C
	Type I IFN Reponse	MX1,MX2,ISG20,DDX4
	Type II IFN Reponse	GPR146,SELP,AHR
	HLA	HLA-A,HLA-B,HLA-C,HLA-E,HLA-F,HLA-G,HLA-H,HLA-J,HLA-L,HLA-DMA,HLA-DMB,HLA-DOA,HLA-DOB
	CT	MAGEA2,MAGEB2,MAGEC2,PAGE4,PRAME,CTAG1B
	immune cell infiltration	FOXP3,CXCR5,CD68,CD247,CD8A,PTPRC,MS4A1,CD1A
	Treg	BCL11B,CD4,CCR8,FOXP3,MSLN,IL2RA,VTCN1
	immune checkpoint	CTLA4,LAG3,IDO1,IDO2,TIGIT,BTLA,CD274,PDCD1LG2,PDCD1
	TILs	CD2,CD6,CD8A,CD79A,CD247,CYBB,SELL,STAT4
	CCR	CCR1,CCR2,CCR3,CCR5,CCR7,CCR8,CCR9,CSF2
	metastasis-promoting	SPNS2,GRSF1,C17orf62,CYBB,FAM175B,BACH2,NCF2,ARHGEF1,FBXO7,TBC1D22A,ENTPD1,LRIG1,CYBA,HSP90AA1,NBEAL2
	metastasis-inhibiting	IRF1,RNF10,PIK3CG,ATPBD4,SLC9A3R2,IRF7,FAM108A1
	pro-inflammatory	STAT1,GZMB,CD19,CD8B,GNLY,IFNG,IL12A,PRF1
	parainflammation	AIM2,CD14,CD276,HMOX1,LGMN,MX2,MMP7,TLR2

Table S2

Variable		#pz(%)
**Age**	age<=53(Q1)	139(27%)
	53<age<69(Q1-Q3)	250(48%)
	age>=69(Q3)	130(25%)
**Sex**	Female	136(26%)
	Male	384(74%)
**T**	T1	35(7%)
	T2	151(29%)
	T3	135(26%)
	T4	183(35%)
**N**	N0	244(47%)
	N+	258(50%)
**Stage**	StageI	20(4%)
	StageII	98(19%)
	StageIII	105(20%)
	StageIV	276(53%)
**HPV**	Neg	421(81%)
	Pos	97(19%)
**Smoking history**	No smokers	117(22%)
	Smokers	177(34%)
	Reformed smokers (>15years)	73(14%)
	Reformed smokers (<15years)	139(27%)
**Alchol consumption**	No	162(31%)
	Yes	347(67%)
**TP53 mutation**	WT P53	152(30%)
	MUT P53	360(70%)
**CDKN2A mutation**	WT CDKN2A	400(78%)
	MUT CDKN2A	112(22%)
**FAT1 mutation**	WT FAT1	399(78%)
	MUT FAT1	113(22%)
**PIK3CA mutation**	WT PIK3CA	419(82%)
	MUT PIK3CA	93(18%)

Table S3

	HNSCC, average Immune signatures					
	**Estimate b**	**OR[CI95%]**	**pval**	**Rsquare**	**adjRsquare**	**model pval**
				0.055	0.047	1.1E-05
**HPV+ vs HPV-**	0.14	1.15[0.95-1.38]	0.15			
**mutP53 vs WT**	-0.23	0.79[0.67-0.92]	0.003			
**Smokers vs no Smokers**	-0.08	0.92[0.81-1.06]	0.25			
**Male vs Female**	-0.19	0.83[0.71-0.96]	0.016			
**T3T4 vs T2T1**	-0.09	0.91[0.80-1.05]	0.19			
