# Supplementary material for: Immunosignatures associated with TP53 status and co-mutations classify prognostically head and neck cancer patients
Source: Mol Cancer. 2023 Nov 30;22:192. doi: 10.1186/s12943-023-01905-9 (PMC10687972; doi:10.1186/s12943-023-01905-9)
Supplement: Supplementary file 2 — Additional file 2: Table S1. List of the immune gene sets analysed. This table comprises the immune gene sets analyzed, listing all genes utilized in determining the immune score. The immune score was evaluated through the z-score transformation of the average expression derived from the complete gene list. The genes and their respective correlation with immune activity were obtained from Lyu HY et al (Computational and Structural Biotechnology Journal, 2019). Table S2. Descriptive characteristics of TCGA HNSCC dataset. Table S3. Multivariate regression models of the main clinical variables associated with the immune signatures in HNSCC. The model was built considering a gene signature including all the 125 genes composing the immune gene sets. [file 12943_2023_1905_MOESM2_ESM.docx]

**Methods**

Data were derived from the “The Cancer Genome Atlas” (TCGA - the HNSCC-TCGA, Nature 2015), and the analyses included 520 HNSCC patients. We gathered the normalized TCGA HNSCC gene expression data from Broad Institute TCGA Genome Data Analysis Center (<http://gdac.broadinstitute.org/>): Firehose stddata__2016_01_28 and Broad Institute of MIT and Harvard. [doi:10.7908/C11G0KM9](http://ezid.cdlib.org/id/doi:10.7908/C11G0KM9).

Clinical information for cohorts was collected from cBioPortal (<https://www.cbioportal.org/datasets>), according to the data published by Liu et al. [9].

A second cohort of 108 HNSCC HPV negative patients (Huang et al., PMID: 33417831) was obtained from CPTAC (https://www.linkedomics.org/login.php#dataSource).

Transcriptomic landscape of HN cell lines was obtained from Iorio et al (array express, E-MTAB-3610, PMID: 27397505).

To identify HNSCC patients responsive to immunotherapy, for each gene set and immune checkpoint protein we initially developed a logistic regression model based on the average expression of the immune signature genes. Specifically, the mean expression values of the genes belonging to the specific immunological signature were used to build linear regression models and to assess their associations with several clinical variables. Odds ratios with confidence intervals at 95% were evaluated for each gene set by including age, gender, tumor size, limphnode status, stage, HPV status, smoking history, TMB, and the mutational status of *TP53*, *PIK3CA*, *FAT1* and *CDKN2A*. Significance was defined at the 5% level (p<0.05). Results are presented as Odds Ratio values (OR) with 95% confidence intervals. The significance of gene/signature modulation between different subgroups of samples was assessed by Wilcoxon test or ANOVA test, as appropriate. The analyses were conducted with Matlab R2022a.

Kaplan-Meier curves of HNSCC patients with high or low immune scores were conducted in order to assess the overall survival (OS) and progression free survival (PFS). Differences between curves were evaluated by the Logrank test. Hazard ratios with 95% confidence intervals were assessed by Cox Hazard regression models. Immune scores were evaluated as the positive and negative z-scores of the average expression of the 125 genes composing the 26 immune gene sets.

To investigate the cellular heterogeneity landscape of the tissue expression profiles, we performed a cell type enrichment analysisusing *XCell* (<https://xcell.ucsf.edu/>), a gene signature-based method to associate gene expression profiles with 64 immune and stroma cell types [10] .

A validation cohort of 102 HNSCC patients treated with PDL1 inhibitors was gathered from GEO database with accession ID GSE159067 [11] .

We also analyzed overall survival based on mutational status using data from Samstein's cohort (MSKCC), which comprises 139 HNSCC patients treated with ICI. You can find the data at https://www.cbioportal.org.

**Cell cultures and transfection**

Cal27, FaDu and Detroit 562 cell lines were obtained from ATCC (Rockville, MD, USA). These cells were cultured in RPMI-1640 (Cal27, FaDu) and DMEM (Detroit 562) medium (Invitrogen-GIBCO, Carlsbad, CA) supplemented with 10% fetal bovine serum and 20% for Detroit 562, penicillin (100 U/mL), and streptomycin (100 mg/mL; Invitrogen-GIBCO). All cell lines were grown at 37°C in a balanced air humidified incubator with 5% CO2. The transfections were performed with Lipofectamine RNAiMax. All experiments were conducted according to the manufacturer’s recommendations. siRNAs were purchased from Eurofins MWG (Ebersberg, Germany) and sequences are as follows: si-SCR: 5′-AAGUUCAGCGUGUCCGGGGAG-3′; si-YAP: 5′-GACAUCUUCUGGUCAGAGA-3′; Si-p53: 5′-GACUCCAGUGGUAAUCUAC-3′.The cells were transfected for 48 hours according to the cell line and the experiments (see results).

**RNA processing and qRT-PCR**

Total RNA from cell lines differently treated or not was extracted by using Trizol Reagent following manufacturer’s instructions (Ambion). cDNA was synthesized according to the manufacturer’s instructions (M-MLV RT kit, Invitrogen). Gene expression was measured by real-time PCR using the FastStart SYBR Green Master Mix (Applied Biosytems) on a QuantStudio 5 (Applied Biosystems). Sequences of qPCR primers are ACTIN Fw: 5′-GGCATGGGTCAGAAGGATT-3′, Rv: 5′-CACACGCAGCTCATTGTAGAAG-3; PD-L1 Fw: 5′-CATCTTATTATGCCTTGGTGTAGCA-3′, Rv 5′-GGATTACGTCTCCTCCAAATGTG-3′′.

**Flow cytometry analysis of PD-L1 surface expression in cell lines**

Representative cell lines (color-coded) were harvested from their cultures and stained with CD274-PE mAb or control Ig for 30 min at 4°C. Surface expression was assessed on single, live cells on the Attune NxT cytometer. Mean fluorescence intensity is shown. The staggered plot depicts cell line expression according their mutational status.
